# Examining the Relationship between COVID-19 Related Job Stress and Employees’ Turnover Intention with the Moderating Role of Perceived Organizational Support: Evidence from SMEs in China

**DOI:** 10.3390/ijerph19063719

**Published:** 2022-03-21

**Authors:** Hongshan Lai, Md Altab Hossin, Jieyun Li, Ruping Wang, Md Sajjad Hosain

**Affiliations:** 1 ISCTE-University Institute of Lisbon, 1649-026 Lisboa, Portugal; laihs@zucc.edu.cn; 2 School of Management and Economics, University of Electronic Science and Technology of China, Chengdu 611731, China; 3 Sichuan Dadao Tongxing Robot Co., Ltd., Chengdu 610000, China; lijieyunfr@sina.com; 4 School of Management, China West Normal University, Nanchong 637002, China; wrpqyi@163.com; 5 Business School, Sichuan University, Chengdu 610065, China; sajjad_hosain@yahoo.com

**Keywords:** COVID-19, job stress, perceived organizational support, turnover intention, small and medium enterprises, employee psychology

## Abstract

The outbreak of COVID-19 has exerted an enormous impact on society, enterprises, and individuals. It has affected the work attitudes and psychology of employees to a certain extent and their job stress (JS) has also augmented accordingly, leading to increased turnover intention (TI). With the survey responses of 720 employees of small and medium enterprises (SMEs) in China as the sample, we studied the impact of COVID-19 related JS and TI with the moderating effect of perceived organizational support (POS). We utilized linear and multiple regression analysis using Windows SPSS 25. The research findings indicated that the JS caused by COVID-19 in the first affected region (Hubei) was significantly stronger than that in other regions (non-Hubei). JS had a significant positive relationship with employees’ TI, while POS had a significant negative connection with employees’ TI. We also identified that POS weakened the positive association between JS and employees’ TI. These findings are expected to be conducive to and conductive for the upcoming theoretical and empirical investigations as the founding guidelines, as well as for managers in formulating effective policies to curb JS, which would ultimately be helpful in reducing TI.

## 1. Introduction

At the beginning of 2020, the rise of a sudden pandemic posed a huge threat to the lives and health of people around the world and brought about significant losses to the global economy [1,2]. As an important force in improving people’s livelihoods, expanding employment, and promoting innovation, small and medium enterprises (SMEs) have contributed nearly 80% of jobs, 70% of innovation, 60% of GDP and 50% of tax revenue in China [3]. However, due to their small scale and limited capability against sudden strikes, such as the impact of the coronavirus 2019 disease (COVID-19), their weak anti-risk ability results in a higher employee turnover rate, thereby making the already struggling production and operation even worse [4].

Without any doubt, the outbreak of COVID-19 has sharply increased the job stress (JS) of employees, which in turn has affected employee turnover intention (TI) [5,6,7,8,9]. The notion of JS as a key research topic in management fields, such as management psychology and organizational behavior, originated in the 1970s [10]. Numerous studies have found that JS can cause significant problems, such as job dissatisfaction, burnout, etc., which ultimately produce a higher turnover rate [11]. On the other hand, He et al. (2020) believe that organizational support has a moderating effect on employee TI [12]. Additionally, Meng et al. (2020) emphasize that organizational justice has a negative impact on TI [13]. Recently, a vast number of scholars have begun to pay attention to the impact of COVID-19 on SMEs and have carried out a number of theoretical and empirical studies. However, many of these existing studies [1,5,11] have focused more on the impact of the pandemic on the supply chain of SMEs and their countermeasures, while very few studies have been conducted on employee TI.

Although COVID-19 related JS may affect employees’ TI, we cannot simply associate JS with TI behavior. However, the mechanism of the moderating effect caused by perceived organizational support (POS) should also be considered. Many previous studies have mainly focused on the impact of work attitude and stress on employee TI, but few of those works have considered the effect of POS or, most importantly, global health disasters or pandemic situations (i.e., COVID-19) on employee TI. A few researchers have identified the positive association between JS and TI, while they have also found that POS can moderate the positive relationship between JS and TI [14]. Another study conducted by Wong et al. (2021) has revealed that US hotel employees perceive environment related (i.e., COVID-19) JS as being significantly different from JS during normal periods [15]. However, such studies concentrating on JS, POS, and TI are still quite rare in the academic domain, particularly in the fields of organizational psychology and organizational behavior.

SMEs are the biggest hub for the creation of jobs within any economy. The sector can also be affected first in the case of a disaster, such as the COVID-19 situation. For any export-oriented countries, such as China, the impact may be much worse [16]. Further, long-term pandemics and lockdowns can create JS for the employees, which leads to TI [17]. However, such a reality has been largely absent in terms of receiving the attention of scholars and academics. As such, there is scope to work on this issue in a way that can enrich academia and contribute to the body of the scientific knowledge base. We strongly believe that this empirical study on SMEs in such pandemic situations can substantially enrich the literature and managerial knowledge guided by realistic and practical outcomes.

Perceived JS at the time of the COVID-19 outbreak in China has been linked with psychological health and emotional wellbeing [17], psychological impact [18], level of knowledge, attitude, practice [19], employees’ commitment [20], and psychological distress [21]. However, we identified that most of the existing studies have been limited to the sources, discovery or diagnosis of stress. Only a few of those investigations have paid attention to the importance of searching for techniques to relieve such stress and reduce TI during the pandemic. Furthermore, we can observe that SMEs have been the worst affected sector during this particular pandemic situation, where a huge loss of jobs has been evident [22,23,24,25]. Therefore, there is a substantial gap regarding research investigations on the impact of COVID-19 related JS on TI, particularly within SMEs. Furthermore, as the first affected region (Hubei Province) of China, the SMEs in Hubei province have been the worst affected sector regarding COVID-19 related JS and TI [16].

The present research is expected to enrich the existing literature domain and managerial decision-making in terms of retaining talented employees as a source of competitive advantages and creating value for enterprises, societies, and humanity as a whole. We propose that once employees are valued and supported by their respective organizations, they will repay their organizations by creating superior value. We anticipate that the empirical outcome of this paper will aid academics, managers, and policymakers in their respective fields of work by providing realistic findings that were identified in this empirical study.

Based on this research gap, this article aims to investigate the impact of COVID-19 related JS on the TI of Chinese SMEs employees. Further, we aim to make a comparative investigation of the JS caused by the pandemic between the first affected region (Hubei Province) and the non-Hubei regions of China. Finally, in light of the research rationale and background, we put forward the following research questions:

**RQ1:** Is there any difference in the job stress produced by COVID-19 between the first affected region (Hubei Province) and non-Hubei areas?

**RO2:** What is the impact of COVID-19 related job stress on employee turnover intention among the employees of Chinese SMEs?

**RQ3:** Can perceived organizational support moderate the relationship between job stress and turnover intention?

The first research question focused on whether there is any significant difference in COVID-19 related JS between the first affected region (Hubei Province) and other regions of China (non-Hubei areas). In order to make a comparison, we selected 42.22% of respondents from Hubei Province and the other 57.77% from non-Hubei regions. The second question aimed to find out whether there is a significant relationship between COVID-19 related JS and TI among the employees of Chinese SMEs. Finally, the third research question explored whether POS can moderate the relationship between JS and TI among the employees of Chinese SMEs.

## 2. Literature Review, Research Hypotheses and Theoretical Framework

### 2.1. COVID-19 and Job Stress (JS)

JS refers to a series of physiological and behavioral reaction processes felt by individuals, which are produced by coercive and threatening factors that are in turn caused by work behaviors within the work environment and under the interaction between the individuals and their work environments [26]. Cooper et al. (1988) put forward the stress system model and occupational stress indicator (OSI) in 1988 and pointed out that the amount of JS is mainly affected by factors including the work itself, the role of the individual within the organization, career development obligation, interpersonal relationships at work, and organizational structure [27]. Based on the previous results, Mattenson and Ivancevich (1987) believed that work stress can be divided into internal stress and external stress, emphasizing the influence of individual differences and individual perceptions on JS [28]. Cavanaugh et al. (2000) posited that JS is related to job safety requirements, such as role conflict, role ambiguity, and job insecurity [29].

According to a recent study conducted by Wang et al. (2020), the sudden pandemic that started at the beginning of 2020 has made Chinese employees nervous, particularly during the early days of the pandemic, which has created a specific kind of mental stress [30]. Jaime et al. (2020) found that the occurrence of COVID-19 has made medical staff feel anxious and stressed [31]. Wang et al. (2020) discovered that although current COVID-19 levels have been alleviated, the demotions, pay decreases and work related anxiety of SME employees that have been caused by COVID-19 and the corresponding difficulties in corporate development still exist, which are likely to continue to cause worry and panic as well as an increase in JS, leading to voluntary resignations from jobs [30]. A survey conducted on the impact of COVID-19 on Chinese SMEs by Dai et al. (2020) during the early periods of the pandemic revealed that 20 percent of the surveyed firms were unable to survive beyond a month on a cash flow basis and 64 percent were not able to survive beyond three months, which presents a dire picture of SMEs bankruptcies under the extended pandemic scenario [32]. Another contemporary study conducted by Sun et al. (2021) has revealed that COVID-19 has significantly affected the mental health, productivity, and performance of Chinese SMEs employees [33].

Survey-based research that was conducted by Tsinghua University on 995 SMEs in February 2020 indicated a decrease of more than 50% in income among 30% of the companies surveyed; another 28% companies reported a 20 to 50% drop. However, in Hubei Province, the scenario was much worse with 55% of SMEs reporting that they had shut down their business due to the quarantines and cash flow problems. Most of the financial pressure (i.e., 62.8%) derived from paying employee insurance, salaries, and social security, whilst rent and loan payments were the second and third largest causes of stress [34].

Although we have factual data on the financial impact of COVID-19 on Chinese SMEs, there is still a dearth of studies investigating the impact of COVID-19 and JS on the employees of Chinese SMEs. Furthermore, we found that no studies so far in academia have compared the impact of perceived COVID-19 related employee JS in the first affected region (Hubei Province) to that in other parts of China. On the basis of previous studies, this empirical paper uses four indicators to measure JS: work intensity, working conditions, interpersonal relationships, and career development. In China, COVID-19 first broke out in Wuhan, Hubei, and then became a pandemic. Individual employees in regions with different severities of the pandemic have experienced very different levels of stress and corresponding responses. Therefore, we proposed the following hypothesis to be tested:

**Hypothesis** **1.** *Job stress caused by COVID-19 in the first affected region (Hubei) is significantly different from that in other regions (non-Hubei).*

### 2.2. Job Stress (JS) and Turnover Intention (TI)

JS, the perceived psychological state of an employee, who feels that they are in an abnormal mental condition, feels anxious, and/or sometimes suffers from burnout [35]. In relevant research, Mazerolle and Maahs (2000) found that excessive JS may cause a series of abnormal reactions and discomforts in employee physiology, psychology, and behavior, which ultimately make employees tend to produce negative emotions, such as anger and depression [36]. In other research conducted on the TI of front-line employees in SMEs that are involved in logistics enterprises, Jie and Fu (2019) identified that workload, working hours, monotonous repetition, interpersonal consumption, and work–family conflict all have a significant positive impact on the TI of employees [37]. Furthermore, Zhang et al. (2015) studied the role of emotional responses to JS on TI, and found that JS has a significant positive predictive effect on TI [38]. On the other hand, however, a recent study conducted by Kim et al. (2020) revealed that JS does not positively affect the TI of the teachers in secondary schools [39].

The large-scale outbreak of COVID-19 has exerted a huge economic impact on SMEs. As a result of the current state of employment affairs, the JS of employees has increased. The outbreak of COVID-19 has made the unstable performance of SMEs more prominent and has sharply increased the JS of the employees, which in turn has caused them to reconsider their future career plans, leading to TI. A very recent study conducted by Al-Mansour (2021) on Saudi healthcare workers revealed that JS caused by the outbreak of COVID-19 has a significant impact on the TI of front-line Saudi healthcare workers who were considering to change their jobs within the next few months [40]. Wang et al. (2020) reported that the current COVID-19 situation has severely affected Chinese SMEs through demotions, pay decreases, and work related anxiety that has ultimately led to employees’ voluntarily resigning from their jobs [30]. Although there is sufficient literature evidence focusing on the relationship between JS and TI, the COVID-19 outbreak and subsequent lockdowns have placed us in a novel circumstance in which such stress can be a significant predictor of TI for employees, particularly SMEs employees as they were the first affected sector of the economy. Moreover, we made the first attempt to empirically identify COVID-19 related JS as a predictor of TI, which is rare not only among Chinese scholars but also within the entire organizational behavior domain. Evidently, however, the number of studies focusing on the impact of COVID-19 related JS on the TI of Chinese SMEs is comparatively lower than that of necessity and therefore, we aimed to test the following hypothesis:

**Hypothesis** **2.** *COVID-19 related JS has a significant positive relationship with employee TI.*

### 2.3. Perceived Organizational Support (POS) and Job Stress (JS)

POS indicates the general belief of employees that their organization respects and values their contributions and is concerned about their welfare [41]. Rhoades and Eisenberger (2002) posited that reductions in employees’ POS may result from the stress that they usually experience within their role [42]. According to George et al. (1993), POS decreases negative physiological and psychological reactions that are caused by JS because employees receive material and emotional support from their organization when coping with high job demands [43]. Empirical studies have also shown that POS negatively relates to burnout [44,45]. According to Rhodes and Eisenberger (2002), four mechanisms proposed by organizational support theory [46] underlie the indirect relationships of three categories of treatments received by employees from their organization: fairness, supervisor support, rewards, and job conditions. They found that these mechanisms lead to POS outcomes, such as increased job satisfaction, positive mood, reduced strain, increased affective commitment, superior performance, and reduced turnover [42].

Due to the long-term emergency situation created by COVID-19 and following job related stress that has been burdened on SMEs employees, we suggest that we need to identify the causes of job stress. Employees consider that many stressors (such as work overload, role ambiguity, and role conflict) could be restricted by their organization, thus reducing employees’ JS through effective POS. A number of previous studies have revealed that POS has a mediating role on the association of stress with anger and depression symptoms [47] and with TI [48,49], which has provided the initial evidence that POS may mediate the relationship between JS and TI.

### 2.4. Perceived Organizational Support (POS) and Turnover Intention (TI)

Eisenberger et al. (1986) put forward perceived organizational theory on the basis of social exchange theory and organizational anthropomorphism, with POS as the core concept of the theory [46]. Organizational support theory emphasizes that the organization’s care and attention toward employees is an important reason for employees to stay in the organization and make contributions to the organization. The proposal of this theory overcame the limitations of previous studies, which emphasized the commitment of employees to their organization but rarely paid attention to an organization’s commitment to its employees [50].

POS can act as a reducer of TI during any distress that falls on an organization and its employees [14,41]. It may be the only remedy that could cure the internal and external stress related anxiety of an individual employee. In a situation such as the COVID-19 outbreak, such support is required to keep talented and competent employees and retain the sources of competitive advantage [41]. Therefore, we believe that there is scope to work on the relationship between POS, JS, and TI in emergency situations, such as the recent COVID-19 outbreak, which are yet less explored among academics, managers, and policymakers alike.

He et al. (2016) found that organizational support can increase employees’ job adaptability and job involvement, thereby reducing TI [51]. Rhoades and Eisenberger (2002) performed a meta-analysis of more than 70 studies on organizational support and concluded that organizational support increases the organizational affective commitment of employees, increases employee job satisfaction and reduces TI [42]. Cropanzano et al.’s (1997) research also proved that there is a positive correlation between organizational support and TI [45]. In a recent investigation conducted on workers in public sports facilities in South Korea, Choi and Noh (2021) found that POS can moderate the relationship between organizational factors, such as JS and TI [14]. They recommended that although COVID-19 has created a high job instability environment, such job instability and JS environments can be minimized through consideration and support for the service workers of the organization, consequently reducing the TI. Based on the literature evidence, we assumed that POS has a negative relationship with TI for the employees of Chinese SMEs.

**Hypothesis** **3.** *POS has a significant negative relationship with employees’ TI.*

### 2.5. COVID-19 Related Job Stress (JS), Perceived Organizational Support (POS) and Turnover Intention (TI)

POS is the degree to which an individual perceives help and support is offered by their organization. When facing the same stressful situation, employees with higher POS have a higher belief in success than employees with low POS, thereby reducing JS and overcoming the various discomforts caused by stressors [52]. Meyer and Smith (2000) found that POS can affect employee TI through the mediating effect of organizational commitment [53]. Liu et al. (2019) posited that factors affecting job satisfaction, such as rewards, organizational management, and occupation satisfaction can reduce the positive association between JS and TI for rural health workers as a means of POS [54]. As well as in the domain of POS research, social support has also been investigated to observe the moderating effect of POS on the association between JS and TI. For example, Fong et al. (2018) found that social support weakens JS effects on TI [55].

Meng et al. (2020) explored another dimension of POS and found that organizational justice partially moderates the predictive effect of work values on TI [13]. These studies have shown that POS has a buffering effect on JS and TI. Therefore, this existing literature provides clues indicating that as POS increases, the effects of JS on TI decrease. During the COVID-19 outbreak, an investigation conducted by Choi and Noh (2021) on the employees of South Korean public sports facilities revealed that POS moderates the relationship between characteristic job factors, such as JS and TI [14], which provides the underpinning basis for this study. Based on the literature evidence, we assumed that POS has a negative relationship with TI for the employees of Chinese SMEs. However, in the tense atmosphere of COVID-19, we believe that more and more empirical investigations should be conducted on the mediating and moderating role of POS in different regions to underpin this domain of academia. Therefore, POS as moderator role, we proposed the following hypothesis to be tested:

**Hypothesis** **4.** *POS weakens the positive relationship between JS and TI.*

### 2.6. Theoretical Framework

**Figure 1:**
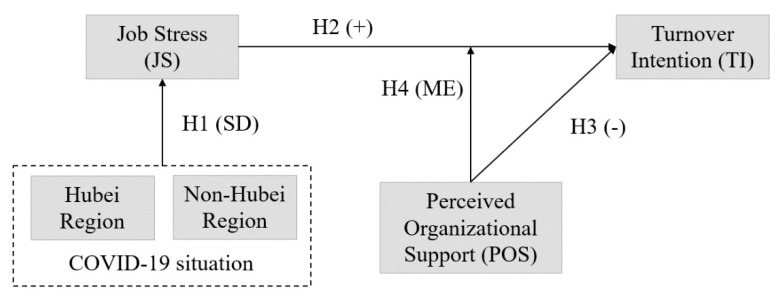
The theoretical framework. Note: SD, significant difference; ME, moderating effect. Source: authors’ own elaboration.

In this empirical study, we considered a single independent variable, JS, a single dependent variable, TI, and a single moderating variable, POS. The proposed theoretical framework is presented in Figure 1.

## 3. Research Method

### 3.1. Sampling Area

We collected primary data in China, 10 months after the outbreak of COVID-19 was first detected. The sample covered employees from the region of Hubei Province, where the pandemic was primarily discovered, as well as respondents who were selected from the provinces of Zhejiang, Henan, Hunan, Sichuan and Guangdong, where the pandemic was relatively severe. We principally targeted the employees of SMEs in those provinces as the sample respondents.

### 3.2. Measurement Tool

As an element of the measurement tool, we used a structured survey questionnaire (presented as Appendix A) to collect data from the respondents utilizing a 7-point Likert scale, where 1 indicated “strongly disagree” and 7 indicated “strongly agree”. The survey instrument had 12 items covering 3 variables (see Appendix A), which were selected from the existing literature. Furthermore, we conducted a confirmatory factor analysis (CFA) to confirm the convergent and discriminant validity among the studied variables and the overall fitness of the measurement model. All of the variables and items were found to be reliable and had sufficient factor loadings and Cronbach alpha values (see Table 2). Due to the geographic distance between the respondents and COVID-19 prevention policies, we sent an electronic version questionnaire via e-mail after they had agreed to participate in the study. The respondents were informed extensively about the concise and well-structured survey items in order to avoid any confusion or complexities in filling out the questionnaires. Further, they were free to contact us at the time of filling out the questionnaire if they perceived any doubt regarding the survey items. After completing the questionnaire and sending it back to us, we provided them with a small token as a gift. This gift token was purposively used to encourage them to fill out the questionnaire properly and accurately. The respondents were strongly assured that the questionnaires would only be used for this research purpose and that their personal identities would be kept confidential. All of these efforts and techniques were purposively used to reduce the non-response rate, which eventually helped us to control the non-response bias [56,57] of our final results.

### 3.3. Sampling Technique and Sample Size

We adopted the purposive sampling technique to select the respondents since this study had a specific purpose to serve. Purposive sampling is suitable when researchers are required to collect a particular type of preferred data from a particular group of people, either since they are the only people who can supply such desired information or they conform to certain criteria set by the researchers [58]. This sampling technique is convenient when researchers use the sample to conform to specific criteria [59]. Therefore, we used purposive sampling to collect data from those respondents who could provide us with sufficient information regarding JS, POS, and TI. Thus, the respondents were selected purposively if they were working for different Chinese SMEs in the selected provinces. This selection of potential respondents was the most suitable for providing relevant and accurate scores for JS, POS, and TI, rather than large numbers of non-relevant respondents who were willing to respond but had a less rationale for the questionnaires, and could eventually prevent biasing by 80–100% [57].

Before the formal survey, we conducted a pre-survey on a small number of respondents to evaluate the rationality and wording of the questionnaire design so as to revise it ahead of time. We initially selected 1000 respondents from the pre-selected Chinese SMEs. However, we received 790 completed questionnaires from them (a response rate of 79%); however, 70 of those were found to be incomplete or incorrectly filled out and were therefore discarded from our consideration. Finally, we considered 720 survey questionnaires (*n* = 720) as the valid sample size. The non-response rate (21%) was not due to people not being willing to respond to this research, but rather because they were busy in managing some critical issues related to the pandemic as well as other issues, such as quarantines, internet facilities, unable to work on certain days, etc. Thus, non-response bias [56] was not an issue for our sample or results.

### 3.4. Demographic Information

Following Table 1 highlights demographic information about the respondents that we considered as valid samples.

According to Table 1, the majority of the respondents (40.55%) belonged to the age group of 18–25 years, followed by the age group of 26–35 years (30%). The percentages of male and female respondents were close. Most of the respondents (44.58%) had job experience of 11–20 years. We purposively selected near equal portions of respondents from Hubei and non-Hubei regions in order to compare the JS levels. Additionally, we found that more than half (54.17%) of the respondents were unmarried and a major portion (55.69%) of them had a bachelor’s degree.

## 4. Analysis and Interpretation

### 4.1. Reliability and Validity Test

The scales used in this paper originated from the measurement scales used in other Chinese and foreign literature. However, in order to ensure the accuracy and reliability of the scales, we conducted a reliability analysis using Cronbach alpha values for each variable. The results of the reliability and validity test using Windows SPSS, Version 25.0 (IBM, Armonk, NY, USA) and Amos, Version 25.0 (IBM, Armonk, NY, USA) are outlined in Table 2.

According to our analysis, the lowest factor loading for the items was 0.610, which is supported by Hair et al. (1998) and Field (2000), who recommend a factor loading of 0.50 for each item to be considered as the threshold for retaining items in order to ensure greater confidence [60,61]. On the other hand, the lowest Cronbach alpha value was 0.659, which was well above the recommended limit (0.60) suggested by Nunnally and Berstein (1994). Therefore, all of the items passed the reliability test [62].

A confirmatory factor analysis (CFA) was carried out to examine the model fitness indices and found that all of the indices were within the acceptable range (Table 3): Chi-squared/DF = 2.752, comparative fit index (CFI) = 0.958, Tucker–Lewis index (TLI) = 0.916, root mean square residual (RMR) = 0.055, goodness of fit index (GFI) = 0.951, adjusted goodness of fit index (AGFI) = 0.919, root mean square error of approximation (RMSEA) = 0.049 and standardized root mean square residual (SRMR) = 0.048.

Convergent and discriminant validity was confirmed through testing and comparing the standard measurements reported by the CFA, such as composite reliability (CR), average variance extracted (AVE), maximum shared variance (MSV), and average shared variance (ASV) [71]. Based on the results from Table 2, CR > 0.7, AVE > 0.5 and CR > AVE were satisfied for each construct. Thus, the convergent validity was confirmed for this study. Discriminant validity was also confirmed because AVE > MSV and AVE > ASV were both satisfied for this study.

A further method for determining discriminant validity was carried out using the heterotrait–monotrait ratio (HTMT), which is generally displayed and tested through the matrix of multi-trait and multi-method. The reported HTMT results, shown in Table 4, depict that all of the maximum values of HTMT were 0.703, which is within the accepted threshold value of 0.85 [72]. Hence, discriminant validity was also further confirmed through the HTMT testing approach.

### 4.2. Correlation Analysis

Previous studies have shown that the different backgrounds of the respondents, such as gender, educational level, working experience and marital status, affect the turnover rate of employees [73]. Topel and Ward (1992) found that there is a negative relationship between job experience and turnover rate [74]. This study treated those individual employee characteristics as control variables. The correlation matrix, mean, and standard deviation of the variables are presented in Table 5.

Table 5 shows that marital status (r = −0.263, *p* < 0.01) and work experience (r = −0.352, *p* < 0.01) present a significant negative correlation with employee TI, while gender (r = 0.445, *p* < 0.01) and educational background (r = 0.608, *p* < 0.01) present a significant positive correlation with employee TI. JS had a significant positive correlation (r = 0.656, *p* < 0.01) with TI. A similar relationship was identified between POS and TI. The hypotheses were initially supported by the correlation analysis.

### 4.3. Tests of Hypotheses

The sample was divided into Hubei and non-Hubei groups according to region. On the premise that the sample conformed to an approximately normal distribution, the independent sample *t*-test method was adopted. The results for the descriptive statistics and the Levene’s test are depicted in Table 6 and Table 7, respectively.

The sample size of non-Hubei regions was 416, with a mean value of 3.06, and the sample size of Hubei was 304, with a mean value of 4.14. Primarily, employees’ JS was different in the different regions. The Sig value corresponding to the F value was less than 0.05, indicating that the variance of the two groups was heterogeneous and that there was a difference. The Sig value corresponding to the *t*-test was 0, indicating that there was a significant difference in JS levels between the two groups, thereby supporting hypothesis 1. The above results indicate that employees’ JS in severely affected regions was higher than that in other regions. This demonstrates that the COVID-19 pandemic generally increased employees’ JS and that they adopted action strategies to seek change.

Finally, we conducted a multiple regression analysis to identify the internal relationship between the variables (control, independent, moderator, and dependent). Table 8 presents the multiple regression analysis between the variables.

The regression analysis results in the above table indicate that the goodness of fit between the data and the model was satisfactory. We further reported the collinearity statistics following the multiple regression analysis to check whether there were multicollinearity issues. Multicollinearity only matters in the presence of an acute relationship between two or more independent variables and thus, dramatically affects the reliability of the multiple regression model because of the inflation in standard errors. It can be tested through two kinds of collinearity statistics: the variance of inflation factor (VIF) and tolerance, which is the inverse of the VIF (i.e., 1/VIF). Through the range of VIF or the tolerance, multicollinearity issues can be identified and corrected. For example, when the range of VIF is from 0 to 5 or tolerance less than 0.2, it is treated as weak multicollinearity; when the range of VIF is 5 to 10 or the tolerance is 0.2 to 0.1, it is treated as moderate multicollinearity; when the range exceeds previous two ranges, it is treated as strong multicollinearity [61]. From Table 8, it can be observed that our reported VIF values were between 1.254 and 2.38 and the tolerance was between 0.798 and 0.430, which indicates that the multiple regression outputs were not biased through collinearity issues.

According to the results of the multiple regression analysis, JS had a significant positive relationship (model 2, β = 0.80, *p* < 0.001) with the dependent variable, TI, thereby supporting the second hypothesis. On the other hand, POS had a significant negative relationship (model 3, β = −0.33, *p* < 0.001) with TI, which supports our third hypothesis.

**Figure 2:**
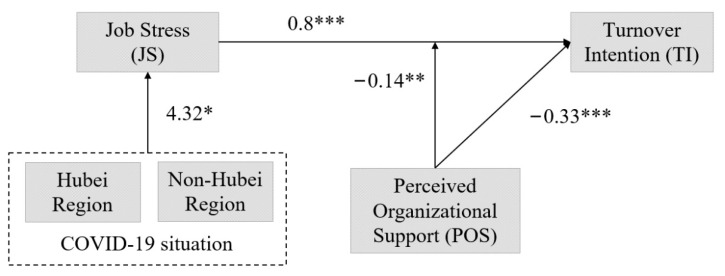
The theoretical framework with the hypotheses testing results. Source: authors’ own elaboration. Note: * *p* < 0.05; ** *p* < 0.01; *** *p* < 0.001.

**Figure 3:**
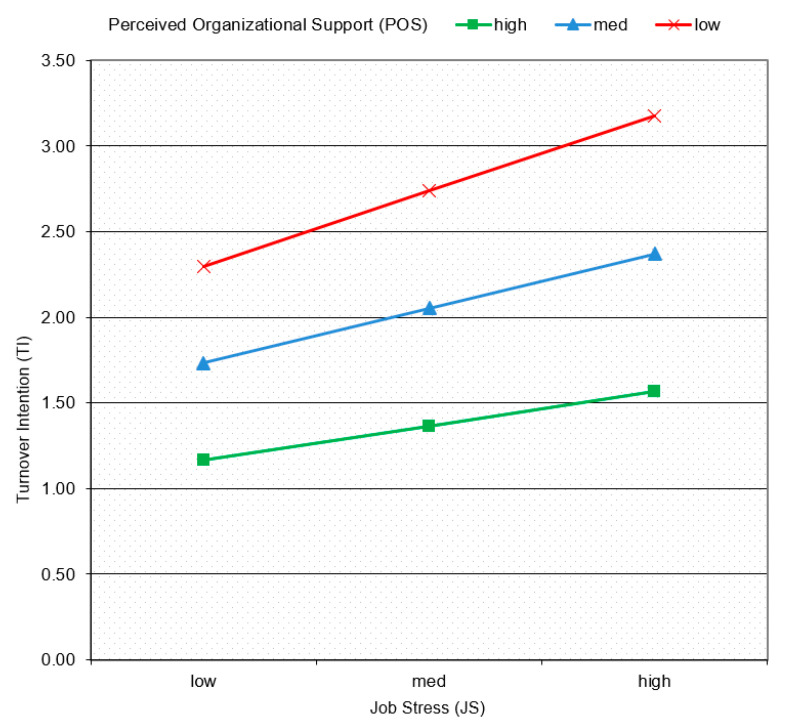
The interaction effects of POS on JS and TI.

The results from the hierarchical regression analysis revealed that the interaction between JS and POS had a significant declining effect on employee TI (model 4, β= −0.14, *p* < 0.01). Figure 2 displays the theoretical framework along with the hypotheses testing results. Moreover, from the interaction effects displayed in Figure 3, it is also clear that POS reduced the intensity of the impact of JS on TI. This indicates that the higher the POS, the weaker the positive relationship between JS and TI. Therefore, our fourth proposed hypothesis (POS weakens the positive relationship between JS and TI) is also supported by the above-mentioned analytical evidence.

## 5. Discussion of Findings

Our analyses revealed that COVID-19 related JS was higher in the first affected region (Hubei Province) than in other provinces. This outcome is quite common, as we learned from the existing literature [26,27,28,29] that has argued such internal and external factors, such as pandemics, political crises, and other natural or artificial disasters, influence the safety and security of job performance due to role ambiguity and perceived insecurity. Many studies have been conducted recently on the role of the COVID-19 pandemic in JS [30,31,32,33,34], which have also produced similar findings.

This study found that JS has a significant positive relationship with employee TI, which is also supported by a number of behavior and human resource management scholars, such as [30,36,37,38,39,40], who have reported that the recent pandemic has created excessive JS, leading to a series of abnormal reactions and discomforts in the physiology, psychology, and behavior of SMEs employees and ultimately leading to them leaving their jobs. It is noteworthy and interesting to mention that most of these studies were conducted from the Chinese perspective, as the highly concerning country of the pandemic along with big population and fast rising of economy. We also found that POS has a significant negative relationship with employee TI, which is also duly supported by a number of Chinese and foreign scholars [14,42,45,46,50,51], who have mostly reported that, based on their empirical findings, proper organizational support from the company and managers reduces tension and internal burnout with positive direction, thus decreasing the chances of employees wanting to leave the organization.

Finally, from our robust and rigorous statistical analyses, we identified that POS weakens the positive relationship between JS and TI. This finding is also somewhat consistent with [14,52,53,54]. Those authors believe that POS can positively reduce TI, as well as weaken the relationship between the JS and TI of general employees. Therefore, our findings are mostly consistent with previously reported results, revalidate this study’s proposed hypotheses within the context of Chinese SMEs and answer all of the research questions that were presented earlier in this paper.

## 6. Implications for Theory and Practice

### 6.1. Theoretical Implication

The current study indicates that JS has been higher in the areas that were severely affected by COVID-19 than that in other areas. It further revealed that JS has a strong positive relationship with employee TI, while POS has a significant negative correlation with TI. Finally, it also identified that POS weakens the positive relationship between employee JS and TI.

The examined results of this study can guide upcoming theoretical and empirical investigations focusing on JS, POS, and TI from the pandemic perspective. It has been generally recognized by scientists that the pandemic and its aftermath are not going to end very soon. Although the studies focusing on JS and TI are abundant, investigations focusing on these factors from the perspective of emergency situations, such as pandemics or war, are quite rare in academia. Therefore, the academic domain requires more studies that emphasize the relationship between pandemics (such as the COVID-19 outbreak and subsequent lockdowns) and work-based psychological wellbeing (i.e., job related stress and TI of employees). We strongly believe that future studies can obtain useful aid from our study and also consider its limitations as a study gap.

### 6.2. Practical Implication

This study revealed that pandemic related work pressure can be a source of JS among employees, which could lead to voluntary turnover behavior. Stress found among employees during an emergency period, which has become a major concern for modern psychological scholars as it can deteriorate both employee mental health and job performance. This study has practical implications in reducing JS and TI.

The managers and organizational policymakers can try to reduce the sources of employees’ JS in order to ultimately reduce TI during short- and long-term emergency situations (i.e., the present COVID-19 situation). In this regard, organizational support as perceived by the employees can largely heal perceived stress and reduce TI. Many previous studies have identified that only support from the organization and top-level managers could effectively augment employees’ morale and confidence in such circumstances. Therefore, we strongly believe that reductions in JS and, subsequently, TI can only be brought about by effective and emotional measures from top level management, who can communicate, guide, motivate, and implement those supportive measures in order to reduce JS and TI.

## 7. Limitations and Further Scope

This study has some obvious limitations that should be recognized as study gaps. First of all, this study only examined SMEs, ignoring larger enterprises. Moreover, we could not incorporate a lot of employees into the study sample because they had already left their jobs. This was unavoidable in a situation where there was a limit on the number of employees to be surveyed, but if follow-up studies are carried out in the future, such limitations can be eradicated. Furthermore, a sample size of 720 may not be adequate as China is a very large country. However, during the pandemic, it was very difficult to select the right respondents and collect data from them due to the severe restrictions that were imposed. We propose future researchers to consider a higher sample size for upcoming research investigations.

Second, this study was conducted within the context of COVID-19 and although it was possible to reflect the impact of job instability experienced by SME employees, it was not possible for us to compare the results to the commercial situation. Employment opportunities in Chinese SMEs have increased due to the stabilization of the COVID-19 situation and as a result of the sufficient supply of vaccines. If an additional survey is conducted on the subject and a longitudinal study is conducted by integrating with the data of this study, more in-depth results will be obtained.

Finally, the study was conducted on a specific country (China) and specific organizations (SMEs). Therefore, the study perspective was restricted to a particular culture. However, future studies should be conducted on a cross-country basis to provide wider and more sophisticated results for academia. We also recommend that longitudinal studies be initiated by interested researchers on these issues and perspectives.

## 8. Conclusions and Recommendations

As a source of JS, the COVID-19 pandemic has played a detrimental role in the economy, particularly for SME employees in Hubei Province. JS in Chinese SMEs forces employees to think about leaving their organizations. According to our analytical findings, POS can reduce employees’ intention to leave their jobs. Due to COVID-19, the job instability of workers in SMEs increased significantly and the perceived stress can eventually develop into the intention to change jobs. Proper and solid organizational support is essential for a sustainable working environment, not only in a pandemic but in any circumstance. However, in a pandemic situation such as the present COVID-19 outbreak, such support is more significant in retaining intellectual talents who can be the source of competitive advantage for organizations.

We recommend that top management should frequently communicate with their employees and reassure them that their job is secure and provide a normal pay scale and environment. Such intimate communication between the employees and top level management can bring a sense of confidence among the employees regarding their concerns. Furthermore, we advise managers and policymakers not to increase workloads or cut pay in such emergencies because they can create dissatisfaction and internal burnout among the employees. The employees who leave their organizations are less likely to come back after the emergency period is over. Thus, it is better to retain existing talented employees than hire new ones and investing money into those new employees as training and development costs.

At the end of this study, we can conclude that if the management observes signs of JS among their employees and if such stress is affecting their behavior, it should be controlled and reduced effectively through motivating and supportive organizational policies and measures. Such support can be provided in a number of ways, such as effective communication, proper counseling, incorporating the suggestions put forward by employees, aligning the goals of employees with the overall organizational goals, and caring about the wellbeing of employees. Human Resource department and personnel must implement supportive commitments so that the ideals of the enterprise and the deeds of its employees are congruent in order to ensure a consistent flow of trained and satisfied workers in the future.

The present study emphasizes that organizational policymakers should take on a major role in identifying the sources of pandemic related JS and TI for employees. Being a source of competitive advantage, employees can repay the support of psychological and managerial contributions once the pandemic is over. The payback could be the higher loyalty of the employees toward the organization, thereby contributing to superior creativity and innovation efforts. Since more natural and artificial emergencies may occur in the future, organizations must devise and implement policies that can counter psychological pressures on their employees. Thus, the findings of this study can form initial guidelines for academics as well as managers. Future researchers should consider the research gaps and more empirical and theoretical studies should be conducted that could remove negativities among employees.

COVID-19 is a worldwide emergency that is affecting the physical, psychological, and economical status of almost all countries. Therefore, it is only us who can remove the immediate and enduring effects of this disaster through our well-guided plans and effective implementation policies. In this situation, organizational support can be the core remedy to stabilizing organizational order as well as retaining the sources of competitive advantages.

## Figures and Tables

**Table 1:** The demographic information of the respondents.

Particulars	Options	Frequency	Percent
Age (Years)	18–25	292	40.55
	26–35	216	30.00
	36–45	102	14.17
	46–60	110	15.28
	Total (N)	720	100
Gender	Male	380	52.78
	Female	340	47.22
	Total (N)	720	100
Job experience (Years)	5–10	132	18.33
	11–20	321	44.58
	21–30	267	37.08
	Total (N)	720	100
Location	Hubei	304	42.22
	Non-Hubei	416	57.77
	Total (N)	720	100
Marital status	Married	330	45.83
	Unmarried	390	54.17
	Total (N)	720	100
Educational level	College and below	220	30.56
	Bachelor’s	401	55.69
	Master’s and above	99	13.75
	Total (N)	720	100

Source: survey instrument.

**Table 2:** The reliability and validity analysis of the measured variables.

Variables	Items	Factor Loading	Cronbach Alpha	CR	AVE	MSV	ASV
JS	JS1: During the pandemic, my workload increased and I often needed to work overtime.	0.631	0.763	0.834	0.503	0.430	0.214
	JS2: During the pandemic, I was overloaded with increased responsibilities.	0.799					
	JS3: During the pandemic, it became difficult to communicate with my colleagues and supervisors, making the relationship tense, often without support or sympathy.	0.688					
	JS4: During the pandemic, the company’s training and learning opportunities within the team were reduced and I felt that there were fewer opportunities to improve myself.	0.752					
	JS5: During the pandemic, I often worried about my work performance.	0.663					
TI	TI1: Due to stress, I am tired with my current job and would like to change it if I get a better opportunity.	0.669	0.713	0.821	0.607	0.430	0.187
	TI2: I hope to have a better job than my present job.	0.773					
	TI3: In the next six months, I expect to resign from my present job.	0.881					
POS	POS1: During the pandemic, my organization did not reduce my salary.	0.672	0.659	0.800	0.504	0.073	0.057
	POS2: My organization took care of us during the pandemic.	0.610					
	POS3: My organization did not terminate any employees during the pandemic.	0.729					
	POS4: I am satisfied with the support my organization provided me during the pandemic.	0.813					

Note: JS, job stress; TI, turnover intention; POS, perceived organizational support; CR, composite reliability; AVE, average variance extracted; MSV, maximum shared variance; ASV, average shared variance. Source: SPSS 25 and AMOS 25.

**Table 3:** The model fit indices and their acceptable thresholds.

Goodness of Fit Index	Value	Level of Acceptance	References
Chi-squared/DF	2.752	<5.0	Marsh and Hocevar [63], Hooper et al. [64]
CFI	0.958	>0.90	Hu and Bentler [65], Hooper et al. [64]
RMR	0.055	<0.08	Hu and Bentler [66], Hooper et al. [64]
GFI	0.951	>0.90	Joreskog and Sorbom [67], Hooper et al. [64]
AGFI	0.919	>0.85	Anderson and Gerbig [68], Hooper et al. [64]
RMSEA	0.049	<0.08	Browne and Cudeck [69], Feinian et al. [70]
SRMR	0.048	<0.08	Browne and Cudeck [69], Feinian et al. [70]

Note: DF, degrees of freedom; CFI, comparative fit index; RMR, root mean square residual; GFI, goodness of fit index; AGFI, adjusted goodness of fit index; RMSEA, root mean square error of approximation; SRMR, standardized root mean square residual. Source: SPSS 25 and AMOS 25.

**Table 4:** The HTMT analysis for discriminant validity.

	JS	TI	POS
JS			
TI	0.703		
POS	0.216	0.106	

Note: JS, job stress; TI, turnover intention; POS, perceived organizational support. Source: descriptive statistics (SPSS 25).

**Table 5:** Descriptive statistics (correlation matrix, mean, and standard deviation) (*n* = 720).

Control Variables	Gender	Marital Status	Educational Background	Work Experience	Job Stress	Perceived Organizational Support	Turnover Intention
Gender	1						
Marital status	−0.263 **	1					
Educational background	0.125	−0.173 *	1				
Working experience	−0.352 **	0.610 **	−0.309 **	1			
Job stress	0.389 **	−0.225 **	0.640 **	−0.473 **	1		
Perceived organizational support	0.290 **	0.140	0.158 *	0.077	0.270 **	1	
Turnover intention	0.445 **	−0.307 **	0.608 **	−0.572 **	0.656 **	0.209 **	1
Mean value	1.61	1.22	2.23	1.90	3.48	3.56	3.25
Standard deviation	0.19	0.41	0.71	1.18	0.99	0.88	1.23

Note: * *p* < 0.05; ** *p* < 0.01. Source: descriptive statistics (SPSS 25).

**Table 6:** The descriptive statistics for job stress in Hubei and non-Hubei regions.

Test Criteria	Region	N	Mean	Std. Deviation	Std. Error Mean
Job stress	Non-Hubei	416	3.0618	0.92464	0.08585
	Hubei	304	4.1430	0.68226	0.07931

Source: descriptive statistics (SPSS 25).

**Table 7:** The Levene’s test for the equality of variances and the *t*-test for the equality of means.

Test Criteria		F	Sig.	T	Df	Sig. (2-Tailed)	Mean Difference	Std. Error Difference	95% Confidence Interval of the Difference	
									Lower	Upper
Job stress	Equal variances assumed	4.320	0.039	−8.663	718	0.000	−1.08124	0.12481	−1.3274	−0.835
	Equal variances not assumed			−9.251	717.161	0.000	−1.08124	0.11688	−1.3118	−0.850

Source: SPSS 25.

**Table 8:** The multiple regression analysis.

Variables		TI				Collinearity Statistics	
		Model 1	Model 2	Model 3	Model 4	Tolerance	VIF
Control	(Constant)	0.79 *	−0.10	0.28 *	0.30 *		
	Gender	0.69 ***	0.28 **	0.48 ***	0.49 ***	0.710	1.409
	Marital status	0.23	0.02	0.14	0.11	0.588	1.700
	Educational background	0.82 ***	0.22 **	0.24 ***	0.26 ***	0.533	1.876
	Working years	−0.39 ***	−0.20 ***	−0.14 **	−0.13 **	0.481	2.079
Independent	JS		0.80 ***	0.88 ***	0.82 ***	0.430	2.328
Moderator	POS			−0.33 ***	−0.30 ***	0.735	1.360
Interaction term	JS*POS				−0.14 **	0.798	1.254
	R Square	0.599	0.785	0.841	0.843		
	Adjusted R Square	0.591	0.779	0.836	0.837		
	F	69.18 ***	133.98 ***	161.60 ***	139.36 ***		

Note: * *p* < 0.05; ** *p* < 0.01; *** *p* < 0.001; JS, job stress; TI, turnover intention; POS, perceived organizational support. Source: regression analysis (SPSS 25).

## Appendix A. Survey Questionnaire

**Variables**	**Items**	**Reference(s)**
Job stress (JS)	JS1: During the pandemic, my workload increased and I often needed to work overtime.	[26,27]
	JS2: During the pandemic, I was overloaded with increased responsibilities.	
	JS3: During the pandemic, it became difficult to communicate with my colleagues and supervisors, making the relationship tense, often without support or sympathy.	
	JS4: During the pandemic, the company’s training and learning opportunities within the team were reduced and I felt that there were fewer opportunities to improve myself.	
	JS5: During the pandemic, I often worried about my work performance.	
Turnover intention (TI)	TI1: Due to stress, I am tired with my current job and would like to change it if I get a better opportunity.	[37,38,39]
	TI2: I hope to have a better job than my present job.	
	TI3: In the next six months, I expect to resign from my present job.	
Perceived organizational support (POS)	POS1: During the pandemic, my organization did not reduce my salary.	[41,38,44,38]
	POS2: My organization took care of us during the pandemic.	
	POS3: My organization did not terminate any employees during the pandemic.	
	POS4: I am satisfied with the support my organization provided me during the pandemic.	

## References

[1] World B. The Global Economic Outlook during the COVID-19 Pandemic: A Changed World. https://www.worldbank.org/en/news/feature/2020/06/08/the-global-economic-outlook-during-the-covid-19-pandemic-a-changed-world.

[2] James K.J., Martin A.W., Andres B.S., Rebecca M.N., Karen M.S., Michael D.S. (2021). Global Economic Effects of COVID-19.

[3] Zhang J., Fan Z.Y., Yi X.J., Xi X.C., Zhang Y., Song H., Xu M.D., Li S.Y., Hu X., Tian S. (2020). China’s SMEs Amid the Pandemic: Facing Cash Flow Problems and Awaiting Government Aid.

[4] Lu L., Peng J., Wu J., Lu Y. (2021). Perceived impact of the COVID-19 crisis on SMEs in different industry sectors: Evidence from Sichuan, China. Int. J. Disaster Risk Reduct..

[5] Bautista J.R., Lauria P.A.S., Contreras M.C.S., Maranion M.M.G., Villanueva H.H., Sumaguingsing R.C., Abeleda R.D. (2020). Specific stressors relate to nurses’ job satisfaction, perceived quality of care, and turnover intention. Int. J. Nurs. Pract..

[6] Boudrias V., Trépanier S.G., Foucreault A., Peterson C., Fernet C. (2020). Investigating the role of psychological need satisfaction as a moderator in the relationship between job demands and turnover intention among nurses. Empl. Relat. Int. J..

[7] Chang Y.P., Lee D.C., Chang S.C., Lee Y.H., Wang H.H. (2019). Influence of work excitement and workplace violence on professional commitment and turnover intention among hospital nurses. J. Clin. Nurs..

[8] Chen Y.P., Sang Y., Sun A.Z. (2020). Impact of COVID-19 on enterprise internal control and countermeasures. Financ. Account. Mon..

[9] Hatamizadeh M., Hosseini M., Bernstein C., Ranjbar H. (2019). Health care reform in Iran: Implications for nurses’ moral distress, patient rights, satisfaction and turnover intention. J. Nurs. Manag..

[10] Hargrove M.B., Quick J.C., Nelson D.L., Quick J.D. (2011). The theory of preventive stress management: A 33-year review and evaluation. Stress Health.

[11] Chang K., Lu L. (2009). The influence of occupation on stressors and work behaviours. Int. J. Hum. Resour. Manag..

[12] He J.H., Zuo L., Chang L.J. (2020). Impact of surface acting on employee turnover intention: Mediating role of emotional exhaustion and moderating role of organizational support. Econ. Manag. Sci..

[13] Meng X.L., Chai P.F., Huang Z.W. (2020). Work value, organizational justice and turnover intention and generation gap. Sci. Res. Manag..

[14] Choi S.K., Noh Y. (2021). The Effect of Job Instability and Job Stress on Turnover Intention in the COVID-19 Situation: Focused on the Moderating Effect of Sports Facility Workers’ Perceived Organizational Support. J. Korean Soc. Qual. Manag..

[15] Wong A.K.F., Kim S., Kim J., Han H. (2021). How the COVID-19 pandemic affected hotel Employee stress: Employee perceptions of occupational stressors and their consequences. Int. J. Hosp. Manag..

[16] Lu Y., Wu J., Peng J., Lu L. (2020). The perceived impact of the COVID-19 epidemic: Evidence from a sample of 4807 SMEs in Sichuan Province, China. Environ. Hazards.

[17] Nanjundaswamy M.H., Pathak H., Chaturvedi S.K. (2020). Perceived stress and anxiety during COVID-19 among psychiatry trainees. Asian J. Psychiatry.

[18] Qureshi M.M., Ashraf K.T., Mohsin S., Abdullah Z.M., Ashraf M., Akbar C.A. (2020). The Price of Battling COVID-19: A Cross-Sectional Survey. Pak. Armed Forces Med. J..

[19] Jawed F., Manazir S., Zehra A., Riaz R. (2020). The novel Coronavirus disease (COVID-19) pandemic: Knowledge, attitude, practice, and perceived stress among health care workers in Karachi, Pakistan. Med. J. Islam. Repub. Iran.

[20] Zandi G., Shahzad I., Farrukh M., Kot S. (2020). Supporting Role of Society and Firms to COVID-19 Management among Medical Practitioners. Int. J. Environ. Res. Public Health.

[21] Abid A., Shahzad H., Khan H.A., Piryani S., Khan A.R., Rabbani F. (2020). Perceived Risk and Distress related to COVID-19: Comparing Healthcare versus non-Healthcare Workers of Pakistan. medRxiv.

[22] Harel R. (2021). The Impact of COVID-19 on Small Businesses’ Performance and Innovation. Glob. Bus. Rev..

[23] Siuta-Tokarska B. (2021). SMEs during the COVID-19 Pandemic Crisis. The Sources of Problems, the Effects of Changes, Applied Tools and Management Strategies—The Example of Poland. Sustainability.

[24] Gregurec I., Tomičić Furjan M., Tomičić-Pupek K. (2021). The Impact of COVID-19 on Sustainable Business Models in SMEs. Sustainability.

[25] Hossain M.R., Akhter F., Sultana M.M. (2022). SMEs in COVID-19 Crisis and Combating Strategies: A Systematic Literature Review (SLR) and A Case from Emerging Economy. Oper. Res. Perspect..

[26] Xu X.D., Meng X.B. (2004). Job Stress: Response and Management.

[27] Cooper C.L., Davies-Cooper R., Eaker L.H. (1988). Living with Stress.

[28] Matteson T.M., Ivancevich M.J. (1987). Controlling Work Stress: Effective Human Resource and Management Strategies.

[29] Cavanaugh M.A., Boswell W.R., Roehling M.V., Boudreau J.W. (2000). An empirical examination of self-reported work stress among U.S. managers. J. Appl. Psychol..

[30] Wang J.X., Chen M.Q., Ying X.P., Gao W.J., Tan X.Y., Liu X.L., Wu J. Evolution of Social Mentality within 18 Days in the Epidemic. https://www.sohu.com/a/374146125_186085.

[31] Jaime A.Y., Asghar A.J., Aldo A.R., Li J.Z., Zhang S.X. (2020). Anxiety, Distress, and Turnover Intention of Healthcare Workers in Peru by Their Distance to the Epicenter during the COVID-19 Crisis. Am. J. Trop. Med. Hyg..

[32] Dai R., Hu J., Zhang X. (2020). The Impact of Coronavirus on Chinese SMEs: Findings from the Enterprise Survey for Innovation and Entrepreneurship in China.

[33] Sun Y., Zeng X., Zhao H., Simkins B., Cui X. (2021). The impact of COVID-19 on SMEs in China: Textual analysis and empirical evidence. Financ. Res. Lett..

[34] Bouey J. (2020). Assessment of COVID-19′s Impact on Small and Medium-Sized Enterprises: Implications from China.

[35] Kahn R.L., Smelser N.J., Baltes P.B. (2001). Stress in Organizations, Psychology of. International Encyclopedia of the Social & Behavioral Sciences.

[36] Mazerolle P., Maahs J. (2000). General strain and delinquency: An alternative examination of conditioning influences. Justice Q..

[37] Jie J.Q., Fu L.R. (2019). The impact of job demand on the turnover intention of workers at the production line in small and medium logistic enterprises based on investigation on 35 enterprises in Beijing-Tianjin-Hebei regions. China Bus. Mark..

[38] Zhang Z.D., Zhao Y.L., Liu R.H. (2015). Job stress and turnover intention: Mediating effect of emotional response. China Hum. Resour. Manag..

[39] Kim J., Shin Y., Tsukayama E., Park D. (2020). Stress mindset predicts job turnover among preschool teachers. J. Sch. Psychol..

[40] Al-Mansour K. (2021). Stress and turnover intention among healthcare workers in Saudi Arabia during the time of COVID-19: Can social support play a role?. PLoS ONE.

[41] Hossin M.A., Hosain M.S., Frempong M.F., Adu-Yeboah S.S., Mustafi M.A.A. (2021). What Drives Sustainable Organizational Performance? The Roles of Perceived Organizational Support and Sustainable Organizational Reputation. Sustainability.

[42] Rhoades L., Eisenberger R. (2002). Perceived organizational support: A review of the literature. J. Appl. Psychol..

[43] George J.M., Reed T.F., Ballard K.A., Colin J., Fielding J. (1993). Contact with AIDS Patients as a Source of Work-Related Distress: Effects of Organizational and Social Support. Acad. Manag. J..

[44] Xu Z., Yang F. (2021). The impact of perceived organizational support on the relationship between job stress and burnout: A mediating or moderating role?. Curr. Psychol..

[45] Cropanzano R., Howes J.C., Grandey A.A., Toth P. (1997). The relationship of organizational politics and support to work behaviors, attitudes, and stress. J. Organ. Behav..

[46] Eisenberger R., Huntington R., Hutchison S., Sowa D. (1986). Perceived Organizational Support. J. Appl. Psychol..

[47] Richardson H.A., Yang J., Vandenberg R.J., DeJoy D.M., Wilson M.G. (2008). Perceived organizational support’s role in stressor-strain relationships. J. Manag. Psychol..

[48] Kim A., Mor Barak M.E. (2015). The mediating roles of leader–member exchange and perceived organizational support in the role stress–turnover intention relationship among child welfare workers: A longitudinal analysis. Child. Youth Serv. Rev..

[49] Villanueva D., Djurkovic N. (2009). Occupational stress and intention to leave among employees in small and medium enterprises. Int. J. Stress Manag..

[50] Xu X.F., Che H.S., Lin X.H., Zhang X.M. (2005). Organizational support theory and its research. Psychol. Sci..

[51] He H., Huang Y. (2016). The influence of organizational socialization tactics on millennial newcomers’ proactive behavior and work adjustment: Based on a mediated moderation model. Rev. Econ. Manag..

[52] Cohen S., Wills T.A. (1985). Stress, social support, and the buffering hypothesis. Psychol. Bull..

[53] Meyer J.P., Smith C.A. (2000). HRM Practices and Organizational Commitment: Test of a Mediation Model. Can. J. Adm. Sci./Rev. Can. Sci. L’administration.

[54] Liu J., Zhu B., Wu J., Mao Y. (2019). Job satisfaction, work stress, and turnover intentions among rural health workers: A cross-sectional study in 11 western provinces of China. BMC Fam. Pract..

[55] Fong L.H.N., Chui P.M.W., Cheong I.S.C., Fong D.K.C. (2018). Moderating effects of social support on job stress and turnover intentions. J. Hosp. Mark. Manag..

[56] Cheung K.L., ten Klooster P.M., Smit C., de Vries H., Pieterse M.E. (2017). The impact of non-response bias due to sampling in public health studies: A comparison of voluntary versus mandatory recruitment in a Dutch national survey on adolescent health. BMC Public Health.

[57] Gustavson K., Røysamb E., Borren I. (2019). Preventing bias from selective non-response in population-based survey studies: Findings from a Monte Carlo simulation study. BMC Med. Res. Methodol..

[58] Sekaran U., Bougie R. (2010). Research Methods for Business: A Skill-Building Approach.

[59] Blumberg B., Cooper D.R., Schindler P.S. (2011). Business Research Methods.

[60] Hair J.F., Anderson R.E., Tatham R.L., Black W.C. (1998). Multivariate Data Analysis.

[61] Field A. (2009). Discovering Statistics Using SPSS.

[62] Nunnally J.C., Bernstein I.H. (1994). Psychometric Theory.

[63] Marsh H.W., Hocevar D. (1985). Application of confirmatory factor analysis to the study of self-concept: First-and higher order factor models and their invariance across groups. Psychol. Bull..

[64] Hooper D., Coughlan J.P., Mullen M.R. (2008). Structural equation modelling: Guidelines for determining model fit. Electron. J. Bus. Res. Methods.

[65] Bentler P.M. (1990). Comparative fit indexes in structural models. Psychol. Bull..

[66] Hu L.t., Bentler P.M. (1999). Cutoff criteria for fit indexes in covariance structure analysis: Conventional criteria versus new alternatives. Struct. Equ. Model. Multidiscip. J..

[67] Jöreskog K.G., Sörbom D. (1993). LISREL 8: Structural Equation Modeling with the SIMPLIS Command Language.

[68] Anderson J.C., Gerbing D.W. (1984). The effect of sampling error on convergence, improper solutions, and goodness-of-fit indices for maximum likelihood confirmatory factor analysis. Psychometrika.

[69] Browne M.W., Cudeck R. (1992). Alternative Ways of Assessing Model Fit. Sociol. Methods Res..

[70] Feinian C., Curran P.J., Bollen K.A., Kirby J., Paxton P. (2008). An Empirical Evaluation of the Use of Fixed Cutoff Points in RMSEA Test Statistic in Structural Equation Models. Sociol. Methods Res..

[71] Fornell C., Larcker D.F. (1981). Evaluating Structural Equation Models with Unobservable Variables and Measurement Error. J. Mark. Res..

[72] Henseler J., Ringle C.M., Sarstedt M. (2015). A new criterion for assessing discriminant validity in variance-based structural equation modeling. J. Acad. Mark. Sci..

[73] Kazi G.M., Zadeh Z.F. (2011). The Contribution of Individual Variables: Job Satisfaction and Job Turnover. Interdiscip. J. Contemp. Res. Bus..

[74] Topel R.H., Ward M.P. (1992). Job Mobility and the Careers of Young Men. Q. J. Econ..

